# Extending the validity of the Feeding Practices and Structure Questionnaire

**DOI:** 10.1186/s12966-015-0253-x

**Published:** 2015-06-30

**Authors:** Elena Jansen, Kimberley M. Mallan, Lynne A. Daniels

**Affiliations:** Institute of Health and Biomedical Innovation, School of Exercise and Nutrition Sciences, Queensland University of Technology, 60 Musk Avenue, Kelvin Grove, Queensland 4059 Australia; Nutrition and Dietetics, Flinders University, Adelaide, SA 5001 Australia

**Keywords:** Non-responsive feeding, structure, restriction, validity, eating behaviour

## Abstract

**Background:**

Feeding practices are commonly examined as potentially modifiable determinants of children’s eating behaviours and weight status. Although a variety of questionnaires exist to assess different feeding aspects, many lack thorough reliability and validity testing. The Feeding Practices and Structure Questionnaire (FPSQ) is a tool designed to measure early feeding practices related to non-responsive feeding and structure of the meal environment. Face validity, factorial validity, internal reliability and cross-sectional correlations with children’s eating behaviours have been established in mothers with 2-year-old children. The aim of the present study was to further extend the validity of the FPSQ by examining factorial, construct and predictive validity, and stability.

**Methods:**

Participants were from the NOURISH randomised controlled trial which evaluated an intervention with first-time mothers designed to promote protective feeding practices. Maternal feeding practices (FP) and child eating behaviours were assessed when children were aged 2 years and 3.7 years **(**n **=** 388). Confirmatory Factor analysis, group differences, predictive relationships, and stability were tested.

**Results:**

The original 9-factor structure was confirmed when children were aged 3.7 ± 0.3 years. Cronbach’s alpha was above the recommended 0.70 cut-off for all factors except Structured Meal Timing, Over Restriction and Distrust in Appetite which were 0.58, 0.67 and 0.66 respectively. Allocated group differences reflected behaviour consistent with intervention content and all feeding practices were stable across both time points (range of *r* = 0.45-0.70). There was some evidence for the predictive validity of factors with 2 FP showing expected relationships, 2 FP showing expected and unexpected relationships and 5 FP showing no relationship.

**Conclusions:**

Reliability and validity was demonstrated for most subscales of the FPSQ. Future validation is warranted with culturally diverse samples and with fathers and other caregivers. The use of additional outcomes to further explore predictive validity is recommended as well as testing test-retest reliability of the questionnaire.

## Background

Parental feeding practices have been shown to influence children’s eating behaviour [1–3] and potentially contribute to the development of childhood obesity by fostering obesogenic eating patterns such as overeating [4,5]. To assess different aspects of these feeding practices, researchers have developed a wide range of measurement tools over the last few decades, each pertinent to a specific research question and scope [6]. As a result, for researchers planning new studies the challenge is not a lack of choice but rather the lack of thoroughly validated, ‘good’ quality measurement tools.

The best indicators for measurement tool quality are the psychometric properties [7,8]. These provide information about the reliability and validity of a tool in a particular sample and provide an indication of the usability of the instrument in other, similar samples. Ideally, psychometric properties should be re-examined to verify usability of the tool in samples that differ (e.g. cross-cultural validity) from that in which the tool was initially developed [6]. A range of psychometric properties are available that can be examined during the steps of questionnaire validation. Ideally a combination of these should be considered, rather than reliance on one (e.g. Cronbach’s alpha). Examples are: 1) Reliability comprising, amongst others, internal consistency (e.g. Cronbach’s alpha), test-retest reliability (correlation between two assessment points), and inter-rater reliability (correlation between two concurrent assessors) [6,9,10]; 2) Validity comprising, amongst others, face validity (clear purpose and good representation of the construct), content validity (experts ensure all aspects of construct are covered and items adequately reflect the measured construct), factorial/structural validity (e.g. Confirmatory Factor Analysis), construct validity (convergent, discriminant; presence or lack of association with related, previously validated measure; or differences between relevant groups), and criterion validity (concurrent and predictive validity; cross-sectional or longitudinal association with ‘gold standard measure’, e.g. observation) [6,9–11]; 3) Sensitivity to change (i.e. level of change following an intervention; difference in scores before/after intervention) [9]; and 4) Stability (i.e. correlation between assessment points, ranking order of individuals remains the same over time) and continuity (i.e. differences between assessment points, mean level of group remains the same over time) [12].

Several recent reviews [6,9,10,13,14] have specifically called for the continued and/or extended validation of questionnaires designed to measure feeding practices. Pinard et al. [6] and Vaughn et al. [9] reviewed the validation of a total of 93 questionnaires and concluded that internal consistency (Cronbach’s alpha) and construct validity were the two most commonly reported psychometric properties. Other psychometric measures such as inter-rater reliability (8 %), factorial (14-25 %) or criterion (3 %) validity, and sensitivity to change (1 paper only) were tested much less frequently [6,9]. Pinard et al. [6] stated that approximately 100 studies did not report any reliability or validity result at all and could not be included in their review. However, of those studies that reported at least one psychometric indicator, in general, the tool showed adequate reliability, while evidence for validity was generally more ambiguous. De Lauzon-Guillian et al.’s review [10] emphasised the need for extended validity testing of existing questionnaires (n = 20, developed for parents with 0-5 year-old children), of which they only found one measure [15] to have been rigorously tested, while 19 tools required further psychometric evaluation.

The Feeding Practices and Structure Questionnaire (FPSQ) is a recently constructed measure of feeding practices related to non-responsiveness and mealtime structure, based on pre-existing items [16] from five questionnaires [15,17–20]. The FPSQ was developed and has been partially validated based on data from first-time Australian mothers enrolled in the NOURISH RCT [21]. The NOURISH trial evaluated an early feeding intervention which focused on increasing responsive feeding practices [22]. Baseline assessment occurred at 4 months of age and outcome assessments when the children were 2 and 3.7 years old. The nine feeding constructs of the FPSQ were theoretically derived and statistically tested, including face validity, factorial validity and internal reliability using the 2-year-old data [16]. Concurrent validity was also established through evidence of cross-sectional correlations with child eating behaviours at 2 years of age [16] reported by mothers via the Children’s Eating Behaviour Questionnaire (CEBQ) [23]. As expected, high levels of non-responsive feeding practices (Distrust in Appetite, Reward for Eating, Reward for Behaviour and Persuasive Feeding) as well as Overt Restriction, and low levels of structure-related feeding practices (Family Meal Setting and Structured Meal Setting) were associated cross-sectionally with higher levels of Emotional Over- and Undereating as well as more Food Fussiness [16].

The overall aim of this paper is to extend the psychometric examination of FPSQ to validate use in both 2- and 3.7-year-old children. Specifically this paper aims to test 1) the factorial validity of the original 9-factor structure at 3.7 years of age, 2) construct validity at 3.7 years of age to the NOURISH intervention [21], 3) the predictive validity of the FPSQ at 2 years using child eating behaviour at 3.7 years of age, and 4) stability of the FPSQ between child ages 2 and 3.7 years. Our hypotheses relating to aims 1-4 are the following:

1. The original 9-factor structure will fit the data when children are 3.7 years of age.
2. In line with intervention content, mothers in the NOURISH intervention group will show lower scores compared to the control group on non-responsive feeding practices but higher scores on structure-related feeding practices.
3. Non-responsive feeding practices will predict higher scores on Food Responsiveness and Emotional Overeating and lower scores on Satiety Responsiveness. Structure-related feeding practices will predict the opposite pattern of associations. Predictive relationships between feeding practices and Enjoyment of Food are ambiguous; while it is considered a food approach variable [23] the ‘face validity’ of the scale might suggest positive connotations.
4. All feeding practices will be stable across both time points.

## Methods

### Participants and procedure

Data were sourced from NOURISH (RCT; Australian and New Zealand Clinical Trials Registry Number 12608000056392); a randomised controlled trial designed to promote feeding practices in first-time mothers that supported healthy child weight and growth by fostering development of healthy food preferences whilst preserving innate capacity to self-regulate food intake [21]. Mothers (≥18 years old) were recruited through maternity hospitals in Adelaide and Brisbane, if they had delivered a healthy term baby (>35 weeks, >2500 g), and had sufficient facility with English to participate in intervention sessions and complete questionnaires. The trial protocol, recruitment and participant characteristics have been described elsewhere [21,24]. Briefly, participants were mailed paper versions of the questionnaires for completion at their convenience. At the 3.7 year assessment time point (SD ± 0.3, range: 3.4-4.2 years), 388 (56 %) mothers (intervention and control group) provided relevant feeding practices data via self-administered questionnaires (see Table 1 for sample characteristics). Fewer participants were included in some analyses due to missing data on maternal feeding practices when children were 2 years old or data on child eating behaviours at 2 or 3.7 years of age. To facilitate analysis with AMOS, missing values on the FPSQ scales were imputed at both time points. No imputations were made for any other variables used in the present analyses. Feeding data from the 2-year-assessment were imputed in a previous study examining the factor structure of the FPSQ [16] and feeding data for the 3.7-year-assessment were imputed here using the same Expectation Maximization (EM) method. Notably, participants missed a maximum of 2 items on the FPSQ at either time point. NOURISH was approved by the Queensland University of Technology Human Research Ethics Committee.

**Table 1:** Sample characteristics (n = 388)

Variable		
Mother	Education level (university degree), % (count)	69 (n = 267)
	BMI (kg/m^2^) at 4 months, M ± SD	25.8 ± 5.2
	Age (years) at 4 months, M ± SD	31 ± 5
Child	Gender (female), % (count)	54 (n = 209)
	BMI z-score (n = 387) at 3.7 years, M ± SD	0.5 ± 0.8

### Measures

#### Maternal Feeding Practices

The 40-item FPSQ [16] assesses nine feeding practices that potentially influence children’s capability to self-regulate their energy intake. Non-responsive feeding practices with a potentially unfavourable impact on the child’s intrinsic capability for intake regulation include: Distrust in Appetite (4 items), Reward for Behaviour (6 items), Reward for Eating (6 items), Persuasive Feeding (6 items), and Overt Restriction (4 items). Feeding practices that potentially support development of autonomy in eating [25] and that are related to the provision of a structured meal environment include: Family Meal Setting (3 items), Structured Meal Timing (3 items), Structured Meal Setting (4 items), and Covert Restriction (4 items). Items were scored from 1 to 5, with higher scores on all feeding practices indicating more frequent endorsement of that practice. The 9-factor-structure of the FPSQ has been previously confirmed via Confirmatory Factor Analysis in this same sample at age 2 years and subscales (based on weighted composite scores) showed internal reliability with Cronbach’s alphas ranging from 0.61 to 0.89 [16]. Mean subscale scores were calculated for analyses.

#### Child Eating Behaviour

Child eating behaviours were assessed using the 35-item Children’s Eating Behaviour Questionnaire (CEBQ) [23]. Since the feeding practices measured with the FPSQ possibly have the ability to influence a child’s capability to self-regulate energy intake [16], only those eating behaviours that clearly reflect self-regulation of intake were chosen: the first food avoidance variable indicates higher ability to self-regulate while the latter three food approach variables indicate lower ability to self-regulate. The four selected subscales were: Satiety Responsiveness (5 items, alpha = .73), Food Responsiveness (5 items, alpha = .75), Emotional Overeating (4 items, alpha = .73) and Enjoyment of Food (4 items, alpha = .88). Mean scores were calculated with a possible range of 1 (lowest) to 5 (highest). The CEBQ has previously shown good psychometric properties (e.g. concurrent validity, internal consistency and test-retest reliability) [23,26] and has been validated in the control group of the present sample at age 2 years (i.e. the factor structure was confirmed and all subscales showed good internal reliability with Cronbach’s alphas between 0.73 to 0.91) [27].

#### Covariates

A range of demographic and anthropometric variables were included in regression models (see below). These included maternal and child age, maternal education level (university degree vs. no university degree) and child gender. Child weight and height were measured by trained study staff [21] and were converted to BMI z-scores (BMIZ) using the WHO Anthro version 3.0.1 and macros [28]. Maternal measured weight and height data from NOURISH baseline assessment (when children were aged 4 months) were used for analysis here to optimise sample size (i.e. 89/349 mothers were pregnant again when children were aged 3.7 years and their exclusion would have decreased the n-value for the regression analyses).

### Data analysis

#### Factorial validity

Confirmatory Factor Analysis (CFA) using maximum likelihood estimation was performed in AMOS 19.0 to confirm the 9-factor structure of the FPSQ [16] when children were aged 3.7 years. The following four goodness-of-fit indices and their respective acceptable cut-offs were used to evaluate model fit [29]: normed chi-square (χ^2^/df; values between 1.0–2.0), Comparative Fit Index (CFI; >0.90), Tucker-Lewis Index (TLI; >0.90), and Root Mean-Square Error of Approximation (RMSEA; <0.08) [30,31]. Cronbach’s alpha with a recommended cut-off value of ≥0.70 was used as indicator of acceptable internal consistency [32].

#### Construct validity

Independent-Samples T-tests were conducted to compare feeding practices, measured when children were aged 3.7 years, of mothers allocated to the NOURISH intervention versus control condition. The intervention group received anticipatory guidance via maternal education and peer support group sessions which were co-led by a dietitian and psychologist [21] and delivered via two modules, each consisting of six 1.5-2 hour interactive group sessions held once every two weeks. Modules commenced when children were 4-7 and 13-16 months of age. Content emphasised the promotion of healthy food preferences (e.g. food exposure), responsive feeding (e.g. appropriate recognition and response to children’s hunger/satiety cues), and positive parenting (e.g. autonomy-encouragement, warmth). Mothers assigned to the control group received ‘usual services’, which was self-directed access to universal child health clinic services such as breastfeeding support and growth monitoring or access to information via a website or telephone help line.

#### Predictive validity

Hierarchical multiple regression analyses were used to examine the association between each feeding practice and each of the four selected eating behaviours (assessed when children were aged 3.7 years) separately, whilst adjusting for covariates. In step 1 we controlled for the covariates child BMIz, gender, age, maternal education level, age, BMI, group allocation and the respective eating behaviour assessed when children were aged 2 years. In step 2 we entered the feeding practice (assessed when children were aged 2 years) as independent variable. Analyses were repeated for the control group only, removing ‘group allocation’ from covariates entered in step 1.

#### Stability testing

In line with similar studies in the area [33,34], Two-tailed Pearson correlations were estimated between feeding practices measured when children were aged 2 and 3.7 years to assess stability across both time points (i.e. consistency in individual’s standing/rank on behaviour over time). Significant moderate to strong correlations (r > 0.40) [35] indicated stability.

## Results

### Factorial validity

Model specifications included those of the original FPSQ model (i.e. one regression weight per factor set to 1, correlations between the nine factors and three error covariances) [16] as well as constraining the three error variances of the Family Meal Setting factor to be equal in order to address the presence of a Heywood case (i.e. negative error variance and standardised regression weight >1) [36,37]. This specified 9-factor model showed acceptable fit: χ^2^/df = 2.27; RMSEA = .06 (Pclose < 0.05), CFI = .85 and TLI = .83. Measures of internal consistency of the 9 subscales are presented in Table 2. All were above the recommended 0.70 cut-off except Structured Meal Timing, Over Restriction and Distrust in Appetite which were 0.58, 0.67 and 0.66 respectively.

**Table 2:** Measures of internal consistency and range of standardised factor loadings of the 9 feeding practices subscales – Australian first-time mothers of 3.7-year-olds (n = 388)

FPSQ subscales	Number of items	Example item	Cronbach’s alpha	Range of standardised factor loadings per subscale
Structured Meal Setting	4	I insist my child eats meals at the table.	0.72	0.53 – 0.71
Structured Meal Timing	3	I decide the times when my child eats his/her meals.	0.58	0.50 – 0.71
Family Meal Setting	3	My child eats the same meals as the rest of the family.	0.86	0.78 – 0.86
Covert Restriction	4	How often do you avoid buying lollies and snacks e.g., potato chips and bringing them into the house?	0.78	0.54 – 0.82
Overt Restriction	4	If I did not guide or regulate my child’s eating, (s)he would eat too many junk foods.	0.67	0.47 – 0.66
Distrust in Appetite	4	How often are you firm about how much your child should eat?	0.66	0.51 – 0.70
Reward for behaviour	6	I reward my child with something to eat when (s)he is well behaved.	0.85	0.61 – 0.81
Reward for Eating	6	When your child refuses food they usually eat, do you encourage him/her to eat by offering a food reward (e.g., dessert)?	0.85	0.57 – 0.79
Persuasive Feeding	6	When your child refuses food they usually eat, do you insist your child eats it?	0.76	0.48 – 0.67

Abbreviation: FPSQ = Feeding Practices and Structure Questionnaire [16]

### Construct validity

Table 3 presents differences in the mean FPSQ subscale scores by group allocation when children were aged 3.7 years. Construct validity testing revealed that 6/9 group differences were significant: mean scores across the 4 non-responsive feeding practices and Overt Restriction were all lower in the intervention compared to the control group, while mean scores of Family Meal Setting where higher (representing eating meals as a family) in the intervention compared to the control group. Structured Meal Setting, Structured Meal Timing and Covert Restriction did not show significant group differences. For the majority of group differences effect sizes ranged from small to medium (i.e. Cohen’s d values ranged from 0.1-0.7, see Table 3) [38].

**Table 3:** Difference in mean (SD) subscale scores by group allocation when children were aged 3.7 years (n = 388) – Construct validity

	Group allocation				
FPSQ subscales	Control (n = 196)	Intervention (n = 192)	Mean difference (95 % CI)	p-value	Cohen’s d
Structured Meal Setting	4.22 (0.62)	4.26 (0.54)	-.03 (-.15, .08)	.588	0.1
Structured Meal Timing	3.80 (0.52)	3.70 (0.58)	.09 (-.02, .20)	.091	0.2
Family Meal Setting	4.18 (0.97)	4.41 (0.91)	-.24 (-.42, -.05)	**.014**	0.3
Covert Restriction	3.32 (0.80)	3.22 (0.80)	.11 (-.05, .27)	.192	0.1
Overt Restriction	3.56 (0.91)	3.32 (0.91)	.24 (.06, .42)	**.009**	0.3
Distrust In Appetite	2.90 (0.66)	2.42 (0.72)	.47 (.34, .61)	**<.001**	0.7
Reward For Behaviour	2.21 (0.75)	1.97 (0.75)	.24 (.09, .39)	**.002**	0.3
Reward For Eating	2.48 (0.66)	2.15 (0.77)	.33 (.19, .48)	**<.001**	0.5
Persuasive Feeding	3.09 (0.61)	2.74 (0.71)	.35 (.22, .48)	**<.001**	0.5

Abbreviation: FPSQ = Feeding Practices and Structure Questionnaire [16]

Items were scored from 1 to 5, with higher scores on all feeding practices indicating more frequent endorsement of that practice.

Significant values (p < 0.05) are presented in bold.

### Predictive validity

As shown in Table 4, 4/9 feeding practices measured when children were aged 2 years predicted children’s eating behaviours at 3.7 years controlling for respective eating behaviours at 2 years. Structured Meal Timing, Family Meal Setting, Covert Restriction, Distrust in Appetite and Reward for Eating were not prospectively associated with children’s eating behaviours. As expected, Structured Meal Setting showed a positive relationship and Overt Restriction a negative relationship with Enjoyment of Food, while Persuasive Feeding and Reward for Behaviour showed a positive relationship with Emotional Overeating. Unexpectedly, using more overt restriction predicted higher Satiety Responsiveness and a more structured meal setting predicted higher Food Responsiveness. The overall pattern of relationships did not differ substantively when repeating analyses using the control group only (data not shown). Of the original 6 significant relationships, 2 were no longer significant at the p < 0.05 level (i.e. Overt Restriction and Enjoyment of Food, Structured Meal Setting and Food Responsiveness), however, changes in beta coefficients were small (<0.02) and the direction of relationships remained the same.

**Table 4:** Relationships between maternal feeding practices measured when children were aged 2 years and child eating behaviours measured at 3.7 years, adjusted for the respective child eating behaviour measured at 2 years (n = 347) and covariates – Predictive validity

	Dependent variables (CEBQ) (standardised betas)			
	Satiety Responsiveness	Emotional Overeating*	Food Responsiveness	Enjoyment Of Food
FPSQ subscales				
Structured Meal Setting	-.012	-.018	**.111**	**.092**
Structured Meal Timing	-.010	.014	-.002	.012
Family Meal Setting	.076	-.032	-.004	-.004
Covert Restriction	.014	-.062	-.050	-.037
Overt Restriction	**.118**	.026	.025	**-.092**
Distrust In Appetite	.001	.074	-.016	-.075
Reward For Behaviour	.072	**.151**	-.006	-.052
Reward For Eating	-.015	.040	.014	-.078
Persuasive Feeding	.001	**.145**	.024	-.086

*Regression model with Emotional Overeating as dependent variable had n = 346.

Abbreviation: CEBQ = Children's Eating Behaviour Questionnaire [23], FPSQ = Feeding Practices and Structure Questionnaire [16]

All hierarchical models were adjusted for the following covariates at first step: child BMI z-score, gender, age, maternal BMI measured when children were aged 4 months, age, education level (university educated vs. not), and group allocation.

Significant relationships (p < 0.05) are presented in bold.

### Stability testing

Table 5 presents Pearson correlations between feeding practices measured when children were aged 2 years and 3.7 years to determine if mothers’ ranking order remained the same over the two time points. All feeding practices were significantly correlated with the respective practice measured at the later assessment point, suggesting good stability in these new measures. Correlations ranged from 0.45 (Overt Restriction) to 0.70 (Distrust in Appetite), indicating that the majority was moderately correlated [35] with measures at the later assessment.

**Table 5:** Bivariate correlations between feeding practices measured when children were aged 2 and 3.7 years (n = 349) – Stability testing

FPSQ subscales	Pearson’ s r*
Structured Meal Setting	.55
Structured Meal Timing	.52
Family Meal Setting	.62
Covert Restriction	.62
Overt Restriction	.45
Distrust In Appetite	.70
Reward For Behaviour	.62
Reward For Eating	.52
Persuasive Feeding	.69

Abbreviation: FPSQ = Feeding Practices and Structure Questionnaire [16]

*All Pearson’s correlations are significant at p < 0.001 (two-tailed).

## Discussion

### Factorial validity

This study extends the validity testing of the FPSQ by reporting additional psychometric indicators across an expanded age range. Confirmatory Factor Analysis of the FPSQ at 3.7 years of age established that the fit of the original 9-factor structure to the data surpassed the desirable level for 1 of 4 fit indices, and approached the desirable level for the other 3 fit indices. Reliability estimates showed good internal consistency on most subscales; 6/9 were above the recommended cut-off value of 0.70 for Cronbach’s alpha. We concluded that the FPSQ showed reasonable factorial validity and reliability in this sample to the extent that the main aims of the study could still be addressed. Having established factor validity, we were able to proceed to demonstrate evidence of construct and predictive validity as well as stability from 2 to 3.7 years of age.

### Construct validity

Construct validity was apparent for 6/9 feeding practices. All group differences were in the expected direction based on the content provided to participants in the intervention arm of the NOURISH RCT [22]. Specifically, the intervention reduced non-responsive feeding practices and Overt Restriction. With respect to the three structure-related feeding practices, an intervention effect was only found for the Family Meal Setting. However, structure-related feeding practices were not a primary focus of the intervention (main focus was on responsive feeding) [22] and mean scores indicate that mothers in both groups already provided considerable structure related to feeding (scores >3.7 out of 5), thus there may have been a ceiling effect. Group differences presented here are in line with findings from the same sample when children were aged 2 years (6 months after receiving the final NOURISH intervention module) [22] using two validated questionnaires (Child Feeding Questionnaire [CFQ] [15] and Parental Feeding Style Questionnaire [PFSQ] [19]) and several study-specific items.

### Predictive validity

Predictive validity was not established for 5/9 feeding practices subscales based on covariate adjusted regression models with a single feeding practice and child eating behaviour as the independent and dependent variables respectively. None of the hypotheses was confirmed for either Structured Meal Timing, Family Meal Setting, Distrust in Appetite, Reward for Eating and Covert Restriction, with evidence from previous studies supporting the latter null-finding [1,16,39]. The following predictive relationships were as expected: (i) Structured Meal Setting predicted enhanced Enjoyment of Food (pleasure of eating together/family meals/social eating) [40–42]; (ii) Overt Restriction was negatively associated with Enjoyment of Food almost 2 years later [43]; and (iii) the non-responsive feeding practices Reward for Behaviour and Persuasive Feeding were positively associated with Emotional Overeating (feeding unrelated to hunger/satiety cues, such as emotional feeding, predicts emotional eating which includes eating for reasons other than hunger) [44–46].

Two feeding practices showed significant predictive associations with child eating behaviours but in an unexpected direction. Firstly, Overt Restriction showed a positive prospective relationship with Satiety Responsiveness contrary to expectations. While it is plausible that Overt Restriction which is non-responsive to a child’s hunger and satiety cues would decrease children’s capacity to self-regulate and hence decrease their satiety responsiveness [47], we found the reverse relationship. One possible explanation is reverse causality. The same unexpected direction of the relationship between Overt Restriction and Satiety Responsiveness was also found with cross-sectional data at both 2 [16] and 3.7 years of age (*r* = 0.17 and 0.22 respectively; p < .05 value; data not shown), as well as cross-sectional data from a large Dutch study (n = 4987) of 4-year-olds [48] assessing restriction to eat with the CFQ [15] (*r* = 0.06, p < .001). Considering that child eating behaviours are stable across time points [33,34] and have a genetic component [49,50], satiety responsiveness in early childhood might not only predict satiety responsiveness later in childhood, but also drive feeding practices such as Overt Restriction later in childhood. Consistent with potential for reverse causality is the finding that higher Satiety Responsiveness measured at 2 years of age also predicted more Overt Restriction at 3.7 years (β = 0.19; data not shown). These findings exemplify the complex bidirectional interactions between feeding practices and child eating behaviours (traits) which appear to be at least in part heritable [49,50].

The finding that Structured Meal Setting predicts a child’s responsiveness to food is also perhaps unexpected and difficult to explain. Food responsiveness, in conjunction with satiety responsiveness, have been interpreted as evidence of a ‘big’ appetite [51] and both are postulated to have a comparatively strong heritability component (between 63-89 %) [49,52]. Our approach to evaluating predictive validity of the FPSQ is consistent with the focus of much of the literature to date that has explored the extent to which maternal feeding practices can influence child weight status, mediated via child feeding behaviour [3,48,53]. However, increasingly it appears that CEBQ is measuring heritable appetitive traits that may well drive maternal feeding behaviours [1]. As such eating behaviours might not have been the ideal variables with which to establish predictive validity for all feeding practices (5/9 factors showed none, 2/9 showed unexpected relationships). This is consistent with three prospective studies in similarly aged children using the CEBQ as outcome measure and showing variable relationships. Bergmeier et al. [54] did not find any significant relationships with the CEBQ (using the CFQ [15] as measure of feeding practices), while Gregory et al. [2] found associations with modelling and pressure to eat but not restriction, and Rodgers et al. [1] found significant relationships with monitoring, emotional feeding and encouragement. The issues of bias and inaccuracies of maternal reporting must also be considered. Future studies should investigate predictive validity, using different, potentially more directly relevant outcome variables, such as observed/experimentally assessed self-regulation of energy intake, children’s food preferences or dietary intake.

### Stability testing

The final aim of this study was to examine stability of the FPSQ subscales in early childhood. Study results revealed good stability in individual ranks of all 9 feeding practices between child ages 2 and 3.7 years (i.e. mothers retain their position relative to others between the two time points). In line with previous research [2,34,55,56], results from the present study show that feeding practices as measured by the FPSQ were, for the majority, moderately correlated [35] between 2 and 3.7 years of age and therefore suggest high levels of stability in these feeding practices during early childhood. Parents seem to have characteristic ways of interacting with their children when it comes to feeding and these start early in childhood and persist over time.

### Strengths and limitations

Limitations of this research include potentially limited generalizability due to the high education level and the primiparous status of the participants. Additionally, all variables (except for weight/height measures) were self-reported by these mothers, at both time points. However, it is unlikely that mothers would remember their previous responses and if any bias occurred, then there is no reason to suggest that this would not be consistent at both assessments (i.e. no limit to reliability). Furthermore, maternal self-report of feeding practices is the most feasible method of assessment (mother is best informant of habitual behaviour), it is more cost-effective, more practical and less intrusive than direct observations and evidence suggests good correspondence between self-report and actual observations of feeding [57]. Missing data were present but the EM method was used to manage these and numbers were low across the FPSQ (maximum 2 items per case*;* 6 % and 4 % of cases had some missing data at 2 years and 3.7 years respectively). Finally, data from both the intervention and control groups were combined for the present study. Using both groups increased the variance within variables of interest, maximised the available sample size and thus reduced risk of type II error. We did not expect patterns of associations to differ depending on group allocation which we were able to confirm with a similar overall pattern of relationships between maternal feeding practices and child eating behaviours when looking at the control group only. Equally, we did not expect any influence of the intervention on the stability measure presented here given that the NOURISH intervention was completed ≥5 months prior to the 2-year assessment with no contact in between.

### Future research

Overall the results reported here support the applicability of the FPSQ as a conceptually-coherent, theoretically-driven and relatively parsimonious measure of early feeding practices related to non-responsiveness and mealtime structure for use in the preschool years.

Future studies need to test validity of the FPSQ across time (i.e. longitudinal measurement invariance) given that results of the CFA on the 2-year-old data [16] were stronger than those reported in the present study when children were 3.7 year of age. Additionally, intervention studies should examine the FPSQ’s sensitivity to change. Before use in different populations (e.g. culturally diverse samples, fathers, or other caregivers, such as childcare workers), further validation work should be conducted and may include cognitive interviewing, adapting terminology of items and/or evaluating the factor structure via confirmatory factor analysis. Design and validation of tools to assess maternal feeding practices require more comprehensive examination of the complex interactions between feeding practices and eating behaviours assessed both directly and by maternal self-report.

## Conclusions

This study extended evidence for validity and reliability of the FPSQ for use in the preschool years. Consistent with data at 2 years of age [16], results showed that the FPSQ has reasonable factorial validity with good reliability of subscales at 3.7 years of age. Scores on subscales of the FPSQ were able to distinguish between participants randomly allocated to an intervention specifically focused on reducing non-responsive feeding practices from those not receiving this intervention. Predictive validity of the FPSQ subscales was assessed through examining prospective associations between maternal feeding practices and child eating behaviours. No evidence for predictive validity was found for 5/9 subscales. Evidence for predictive validity was found for Reward for Behaviour and Persuasive Feeding, and mixed evidence was found for Structured Meal Setting and Overt Restriction. This study also provides evidence for tracking in the magnitude of the FPSQ subscales though early childhood.

## Abbreviations

BMIZ: Body Mass Index z-score
CEBQ: Children’s Eating Behaviour Questionnaire
CFA: Confirmatory Factor Analysis
CFI: Comparative Fit Index
CFQ: Child Feeding Questionnaire
FP: Feeding Practice
FPSQ: Feeding Practices and Structure Questionnaire
PFSQ: Parental Feeding Style Questionnaire
RCT: Randomised Controlled Trial
RMSEA: Root Mean-Square Error of Approximation
TLI: Tucker-Lewis Index
WHO: World Health Organization
